# A new reference genome for *Sorghum bicolor* reveals high levels of sequence similarity between sweet and grain genotypes: implications for the genetics of sugar metabolism

**DOI:** 10.1186/s12864-019-5734-x

**Published:** 2019-05-27

**Authors:** Elizabeth A. Cooper, Zachary W. Brenton, Barry S. Flinn, Jerry Jenkins, Shengqiang Shu, Dave Flowers, Feng Luo, Yunsheng Wang, Penny Xia, Kerrie Barry, Chris Daum, Anna Lipzen, Yuko Yoshinaga, Jeremy Schmutz, Christopher Saski, Wilfred Vermerris, Stephen Kresovich

**Affiliations:** 1 Advanced Plant Technology Program, Clemson University, Clemson, SC USA; 2 Department of Genetics and Biochemistry, Clemson University, Clemson, SC USA; 3 Department of Bioinformatics and Genomics, University of North Carolina at Charlotte, Charlotte, NC USA; 4 Department of Plant and Environmental Sciences, Clemson University, Clemson, SC USA; 5 HudsonAlpha Institute for Biotechnology, Huntsville, AL USA; 6 Department of Energy, Joint Genome Institute, Walnut Creek, CA 94598 USA; 7 School of Computing, Clemson University, Clemson, SC USA; 8 School of Plant Protection, Hunan Agricultural University, Changsha, 410128 China; 9 Department of Microbiology and Cell Science and UF Genetics Institute, University of Florida, Gainesville, FL USA

**Keywords:** Sorghum, Sugar metabolism, Sugar transport, Genomics, Gene expression

## Abstract

**Background:**

The process of crop domestication often consists of two stages: initial domestication, where the wild species is first cultivated by humans, followed by diversification, when the domesticated species are subsequently adapted to more environments and specialized uses. Selective pressure to increase sugar accumulation in certain varieties of the cereal crop *Sorghum bicolor* is an excellent example of the latter; this has resulted in pronounced phenotypic divergence between sweet and grain-type sorghums, but the genetic mechanisms underlying these differences remain poorly understood.

**Results:**

Here we present a new reference genome based on an archetypal sweet sorghum line and compare it to the current grain sorghum reference, revealing a high rate of nonsynonymous and potential loss of function mutations, but few changes in gene content or overall genome structure. We also use comparative transcriptomics to highlight changes in gene expression correlated with high stalk sugar content and show that changes in the activity and possibly localization of transporters, along with the timing of sugar metabolism play a critical role in the sweet phenotype.

**Conclusions:**

The high level of genomic similarity between sweet and grain sorghum reflects their historical relatedness, rather than their current phenotypic differences, but we find key changes in signaling molecules and transcriptional regulators that represent new candidates for understanding and improving sugar metabolism in this important crop.

**Electronic supplementary material:**

The online version of this article (10.1186/s12864-019-5734-x) contains supplementary material, which is available to authorized users.

## Background

*Sorghum bicolor* (L.) Moench is a widely grown cereal crop that has been adapted to a range of habitats and bred for diverse purposes, resulting in drastic phenotypic differences among certain types. Historically, both genetic and phenotypic diversity in sorghum have been driven by its spread throughout the African continent as well as the Middle East, India, and parts of Asia [1], which has resulted in distinct botanical races largely defined by their floral architecture and seed characteristics [2, 3]. Although present-day sorghum genotypes continue to form genetic clusters according to their race and historical geography [3–6], these clusters do not reflect the extent of diversity among modern sorghums, which include morphologically distinct types optimized for different end uses [1], even among closely related individuals of the same race. Understanding the genetic architectures underlying phenotypic differences among types is critical to further crop improvement efforts, but disentangling the signatures of recent and historical selection in order to isolate causative genes can be challenging and will require thoughtful genomic comparisons.

The current reference genome for sorghum is the inbred ‘BTx623,’ a short-stature, early maturing genotype used primarily for production of grain sorghum hybrids. This genotype is phenotypically very distinct from the tall, late maturing sorghums typically grown for stem sugars or high biomass yield [1]. In addition to differences in maturity and grain production, sweet sorghums are most notably characterized by their ability to produce a high concentration of soluble sugars in the stalk, which can be extracted for human consumption [1]. While previous studies have found that changes in transport activity, rather than in sugar synthesis, appear to drive differences in sugar accumulation; the genetic mechanisms underlying these changes have remained elusive using currently available resources [7, 8].

**Fig. 1:**
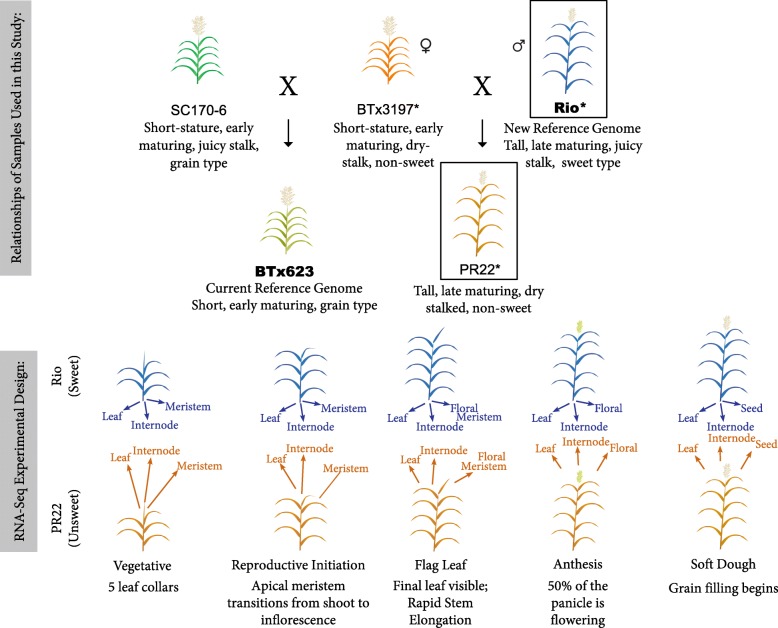
Experimental Design and Relatedness Among Samples. The top portion of the figure depicts the family structure among the lines used for both the genomic and transcriptomic data in this study. Note that BTx3197 is a direct progenitor of both BTx623 and PR22. Orange colored stalks indicate non-sweet, dry stems, while blue stalks indicate sweet and juicy stems. Green colored stalks are intermediate. Bold type denotes lines with a publicly available reference genome. Short read Illumina re-sequencing was performed on any genotype with an asterisk (*) by its name. Boxes show which lines were used in the RNA-seq experiments. The lower portion of the figure shows the 5 time stages and 3 tissues collected at each time point for the RNA-seq study. All images used in this figure were originally created by E. Cooper for this manuscript

To explore all possible genomic differences between sweet and grain types and provide a valuable resource for future studies of sweet sorghum, we generated a second high-quality reference genome by applying Pacific Biosciences long read single nucleotide sequencing to the archetypal sweet line, ‘Rio’, as a contrast to the existing sorghum reference. We also performed comparative transcriptomics on both a temporal and spatial scale between Rio and a non-sweet, recombinant inbred line (RIL) related to BTx623 to capture key changes in both source and sink tissues at different growth stages (Fig. 1).

Genetically, Rio is more closely related to BTx623 than some other sweet sorghum genotypes [9], but exemplifies the striking phenotypic differences that distinguish optimal sweet and grain sorghums. Our results revealed that while there were very few changes in gene content or genome structure between the two sorghum lines, there was a high rate of nonsynonymous polymorphism and a number of genes with complete loss of function mutations. The majority of differences between the lines occurred in genes belonging to large gene families that have undergone extensive expansion in the grasses, including disease resistance genes and a family of transcriptional regulators. Among genes known to be involved in sucrose metabolism, we observed three sucrose transporters that appeared to be either completely deleted or severely truncated in Rio. Several other sucrose transporters as well as some sucrose synthases were differentially expressed between the sweet and grain genotypes, but their changes in expression often did not correspond to any genetic differences within the coding sequence. Many sugar metabolism genes showed altered expression patterns regardless of which allele was present in the RIL, strongly suggesting that they must be regulated by either the presence of sugar, the activity of other genes within the pathway, or upstream regulatory mechanisms. These results highlight the complexity of the genetic interactions driving sugar accumulation in sorghum.

## Results

**Fig. 2:**
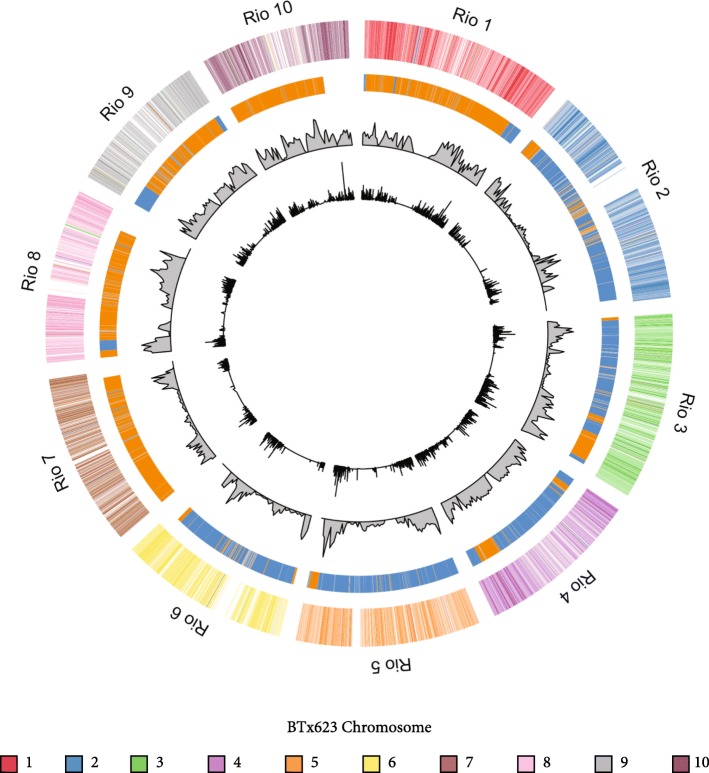
Rio Genome Alignment to the BTx623 Reference. Each segment of the circle corresponds to one of the 10 Rio chromosomes. Segments on the outermost circle are colored according to where they mapped on BTx623. The second ring depicts locations of recombination breakpoints in the RIL (PR22). Blue blocks correspond to segments inherited from the Rio parent, while orange blocks correspond to segments inherited from BTx3197. The third ring the SNP density (number SNPs/200 kb) in 1 Mb sliding windows, while the innermost circle indicates the ratio of nonsynonymous:synonymous mutations in each gene

The chromosome-level assembly of the Rio genome comprised 729.4 Mb, which was 99.6% the size of the BTx623 genome [58]. The amount of repetitive DNA versus gene content was nearly identical, with 35,467 genes identified in Rio versus 34,129 in BTx623. Based on the MUMmer and BLAT alignments, the genomes were largely collinear, with only 2175 rearrangements (Fig. 2). Gene deletions appeared to be more frequent in Rio than gene duplications, even though tandem expansions in general were more common (Additional file 1: Figure S1). Both duplication and deletion events tended to encompass only a single gene at a time (98% of events contained 3 or fewer genes), and mostly within clusters of related genes.

There were 54 genes that appeared to be unique to Rio, which is slightly lower although similar to previous observations based on short read sequencing [10]. While there was no significantly enriched gene ontology (GO) term among these genes, the top GO term was protein kinases. The predicted protein orthologs for each duplicated gene indicated that 12 of these kinases contained nucleotide binding and leucine rich repeat domains (NB-LRR). LRR kinases are commonly associated with disease resistance in sorghum and other plant species [11, 12], so presence/absence in this gene family is likely the result of local adaptation to different disease pressures. These findings are also consistent with the patterns of gene content variation previously observed in diverse rice lineages [13–16].

Of the 276 genes that appear to have been deleted in Rio, NB-LRR kinases were again the top category, with 15 total genes. Another large gene family in plants, the cytochrome P450 superfamily, also appeared to be expanded in BTx623 compared to Rio, with 10 of the deleted genes having orthologs in this group. Cytochrome P450 genes are known to catalyze an extremely diverse set of reactions in plants, so these additional gene copies in grain sorghum could be involved in any number of pathways [17].

The most interesting putative deletions observed in Rio were three known sucrose transporter genes: *SUT4*, *SWEET3–3*, and *SWEET8–2* (Fig. 4 and Additional file 3: Table S1). *SUT4* is one of 6 SUT genes identified in sorghum and its expression has been reported in other sweet sorghum lines, although its exact function and its role in stem sugar accumulation differences may be minimal. [18] The two SWEET genes belong to a larger family of transporters that has a history of expansions in the grasses, with 23 distinct SWEET genes identified in the current sorghum reference genome (Additional file 3: Table S1) [19]. SWEETs are responsible for the transport of sucrose out of the leaf cells and into the phloem, and some members may also be involved in the downstream movement of sucrose from the phloem into the stem storage sink [20]. Previous studies have shown that different SWEET genes are expressed at different times and locations within the developing sorghum plant [21], but the exact function of each gene remains unknown. *SWEET3–3* does not appear to be expressed at all in BTx623 (based on data available within Phytozome), so it is possible that this is not a functional gene copy in sorghum and its deletion in Rio would have little to no effect. *SWEET8–2*, on the other hand, is expressed in BTx623, especially in upper leaves and internodes during floral initiation and anthesis. Its deletion in Rio, therefore, may have significant impacts on the activity of other transporters.

While there are relatively few differences in gene content between the two genotypes, there are a large number of single nucleotide polymorphisms (SNPs) and small insertion/deletion mutations (indels) found throughout the genome (Fig. 2). A total of 1,890,101 SNPs, 167,514 insertions, and 223,952 deletions were identified in Rio with respect to BTx623. The majority of these are located in intergenic or regulatory regions, but for the 98,723 mutations located within a coding region, the overall missense:silent ratio was 1.1, consistent with previous observations in sweet sorghum [10]. A total of 3153 genes exhibited a ratio > 1, with NB-LRR genes once again being the most commonly occurring gene family among them. In addition to these disease resistance genes, there were also two gene families known to have roles in post-translational regulation that show high levels of nonsynonymous mutations: the F-box/RNI-like superfamily and the BTB/POZ-MATH domain containing family. Both of these gene families are known to be highly diverse and fast evolving in plants [22]. F-box proteins are required for a variety of growth- and development-related processes [23], while members of the BTB/POZ-MATH domain family, still relatively uncharacterized in plants, mediate the degradation of various key transcriptional regulators, modulating genes involved in stress response, vegetative growth and stature, as well as floral development [24].

Overall, the majority of within-gene mutations and gene content differences between the sweet and grain genotypes highlight differences in disease resistance, plant growth, and possibly stress response pathways. A handful of structural changes involve sucrose transporters and appear to cause a complete loss of three transporters in the sweet genotype, but it remains unclear exactly how, or if, these deletions affect sugar accumulation.

### Differential expression between sweet and non-sweet Sorghum

A key goal of our comparative transcriptomic study was to not only find changes in expression unique to the sweet genotype, but also to disentangle the effects of changes in plant maturity and development from those related to enhanced sugar accumulation. Sweet sorghums are later maturing than grain types, and floral initiation appears to be tightly linked to the onset of sugar accumulation [8, 25–27]. In order to minimize differences in gene expression that may be related to changes in the maturity times of Rio, we selected an individual that had low Brix (soluble sugar concentration) but with a maturity pattern very similar what is observed for Rio. The RIL used in this study (‘PR22’) is a member of a ‘BTx3197’ Rio mapping population [28], where BTx3197 represents a non-sweet, dry stalked grain sorghum cultivar related to BTx623 [29] (Fig. 1). We also used biological markers, rather than days after planting, to determine when individuals from different genotypes were in the same developmental stage (see Methods for details). We sampled 3 tissues (topmost internodes, topmost fully developed leaves, and either shoot apical meristems, flowers, or seeds) at 5 time points over development, with 3 biological replicates, then sequenced a total of close to 1 billion read pairs on an Illumina HiSeq2500 with standard RNA-seq protocols, and called expression values using standard software.

In both genotypes, Brix values increased linearly over time (Additional file 2: Figure S2), but Rio showed a faster rate of increase and a higher maximum value. The effects of genotype (*p* = 2.2e-16), time (*p* = 2.2e-16) and their interaction (*p* = 1.124e-9) were all statistically significant. Differences in Brix values became significantly different starting at the flag leaf stage (*p* = 4.53e-6), although there were observable differences in the earlier reproductive initiation stage.

**Fig. 3:**
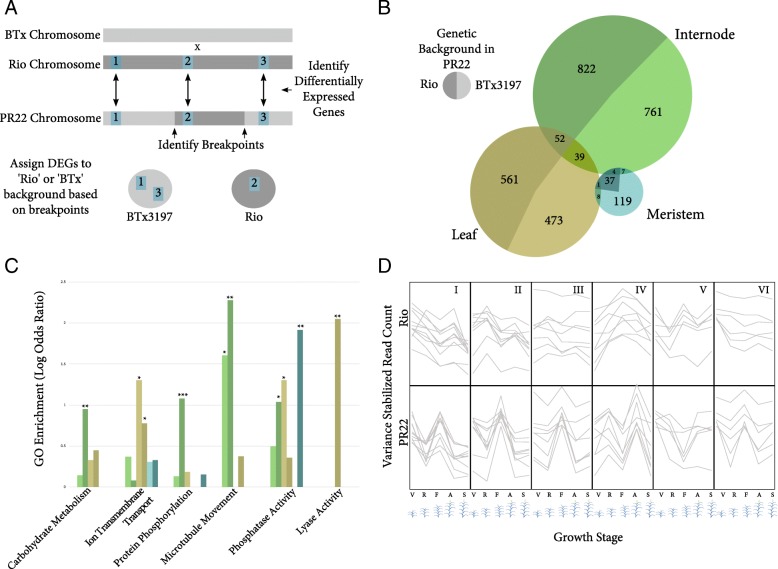
Differentially Expressed Genes in Each Tissue. **a**. This schematic illustrates how significantly differentially expressed genes were assigned to either the ‘Rio’ or the ‘BTx’ background, based on their locations relative to the breakpoints (see also Fig. 1). **b**. A Venn Diagram of DEGs separated by tissue, with each circle of the Venn diagram being further subdivided by how many genes were found on each background. Darker shading indicates genes with a Rio background (i.e. genes that are differentially expressed between the 2 genotypes, BUT had the exact same allele in each), while lighter shading indicates genes with different alleles in the 2 lines. **c**. Significantly enriched GO categories for each tissue type, also subdivided by genetic background. Colors correspond to the categories outlined in panel **b**. Asterisks denote the significance level (* = *p* < 0.01, ** = *p* < 0.001, *** = *p* < 0.0001). **d**. The most commonly observed expression patterns over time. The top row shows expression patterns in Rio, while each graph below shows the corresponding expression pattern in PR22. The x-axis is time (or growth stage), while the y-axis is the variance stabilized count of each transcript

The majority of genes with statistically significant GxT interactions (*p* < 0.001) were differentially expressed in the internode (1686 genes), with slightly fewer differentially expressed genes (DEGs) observed in the leaf tissue (1220 genes), and only a handful found in the meristem (156 genes) (Fig. 3b). After filtering out genes that had identical genetic backgrounds in both genotypes, there were 820 (48.6% of all DEGs) DEGs remaining in the internode, 533 (47%) DEGs remaining in leaf, and 119 (76.2%) DEGs remaining in the meristem. Given that the total percentage of genes found on the BTx3197 background is 47.4%, the likelihood of a gene being significantly differentially expressed in either the internode or the leaf seems independent of genetic background, suggesting that many of these genes are responding to either the increased presence of sugar in Rio or other regulatory signals. Rio begins to deposit sugar in the stems earlier in the growing season compared to the non-sweet sorghum genotypes, so it is inevitable that some changes in gene expression will begin to occur in pathways that utilize sugars as their source. In accordance with this, genes related to carbohydrate metabolism and protein phosphorylation were found to be enriched among DEGs in the internode, but *only* among genes with the Rio allele (Fig. 3c).

Genes related to ion transmembrane transport and microtubule movement were significantly enriched in both the internode and the leaf, regardless of the underlying allele, implying that these genes may have important upstream roles in the sugar accumulation process and simultaneously may also respond to the presence of sugars through some type of feedback loop. Because sorghum, unlike sugarcane, requires an active transport step, transmembrane transporters have previously been implicated as playing a crucial role in the sweet phenotype [30]. The enrichment among genes related to microtubule movement is more surprising, but has also been previously observed. [31] One possibility is that these are involved in the trafficking of key transporters to their correct locations in the cell membrane [32].

Comparing the expression profiles of internode DEGs between genotypes, the most commonly occurring pattern among significant genes was one where PR22 showed increased expression at the flag leaf (FL) stage compared to the other time points, while for the same genes Rio did not show increased expression until one stage later, at anthesis (ANT) (Group I, II, III, and VI in Fig. 3d). Given that the flag leaf stage also marks the time point where Brix significantly increases in Rio, it is notable that most genes would show a delay in expression in Rio rather than an earlier increase and could indicate that Rio increases stem sugar content by *not* metabolizing sugars immediately following the onset of the reproductive phase, but rather delays until after flowering. Several transporters, on the other hand, do show the opposite pattern (see group IV in Fig. 3d), which may indicate their active role in moving sugars into the stem. Of the differentially expressed genes in the leaves, only 12% had any difference in expression at a developmental stage earlier than anthesis, but it should be noted that lower, more mature leaves could be exhibiting different patterns of expression that are not observed in the topmost leaf.

In the meristem tissue, where there are only a handful of significant genes, it is striking that many of them actually still exhibit identical overall expression patterns in both genotypes, but with different slopes (hence the significant GxT terms). A total of 32 (of 156) genes have the same general expression trend, and 10 of these 32 specifically exhibit a pattern of being only upregulated during the flag leaf stage, but down regulated at all other times (Fig. 3d). This is the same pattern that predominates in the internode tissue, but only for the non-sweet PR22 genotype.

**Fig. 4:**
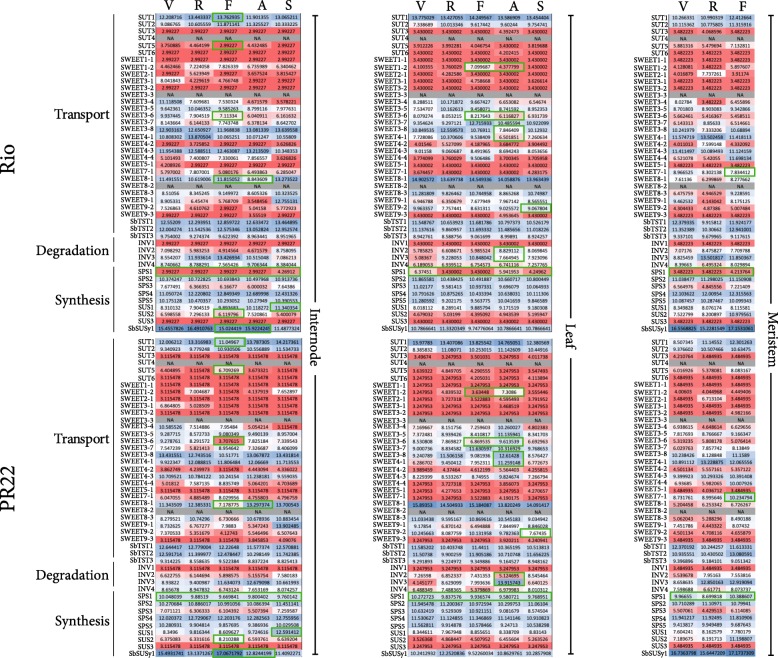
Expression Patterns over Time for Known Sucrose Metabolism Genes. Values inside each table give the variance stabilized count, while colors indicate how much higher (blue) or lower (red) the value is compared to the median value for that gene across both genotypes. Green squares show values with a statistically significant difference between Rio and PR22

Among genes known to be involved in sucrose synthesis or transport, 14 of them have significant expression differences in at least one tissue, but many appear to only be differentially expressed after there are already observable differences in Brix between the 2 lines (Fig. 4). There is a sucrose phosphate synthase gene (*SPS1*) that shows constitutively higher expression in the non-sweet line across all tissues and time points (Fig. 4) despite both genotypes having the same genetic background at this locus. In the internode, four sucrose transporters (*SUT1*, *SUT5*, *SWEET3–6*, *SWEET8–1*) have significant differences in expression at the flag leaf stage, but two of these (*SUT5* and *SWEET8–1*) have the same genetic background in both lines. *SUT1* is more highly expressed in Rio internodes at flag leaf, but the overall patterns of expression for the two lines are similar across time points. While PR22 does have the non-sweet *SUT1* allele, there were no nonsynonymous mutations and only 6 upstream variants in this gene. *SWEET3–6*, on the other hand, has one amino acid substitution (I14V) in a predicted conserved transmembrane domain region (Additional file 8: Table S6 and Additional file 10: Table S8). This transporter is more highly expressed in both internodes and leaves in the sweet line during the flag leaf stage (Fig. 4). We did not observe any significant differences in expression among the tonoplast transporters, in contrast to what has been observed in other studies [7].

Two sucrose synthases (*SUS2* and *SbSUSy1*) also have significant differences in expression during the flag leaf stage, although unlike the transporters they show higher expression in PR22 rather than Rio. Both genotypes have the same *SbSUSy1* allele, but PR22 has the non-sweet allele for *SUS2*. There is a single in-frame insertion in the Rio gene (CGG insertion at position 68,447,685 on chromosome 4) which was predicted to have a moderate impact by snpEff. None of the other differentially expressed sucrose metabolism genes contain any nonsynonymous differences.

**Fig. 5:**
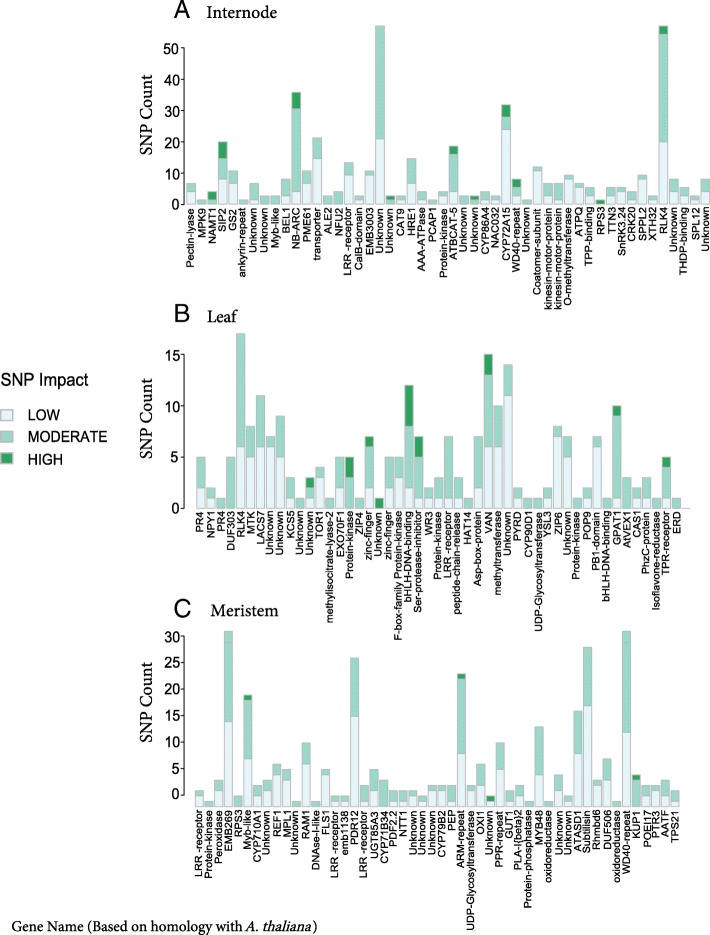
Genes with Missense mutations and significant changes in expression. Within each tissue, DEGs were filtered to contain only genes with different alleles in the comparative expression study, as well as at least one missense mutation. Then, the top 50 DEGs (ranked by the GxT *p*-value) were selected and are plotted in their order of significance from left to right. Gene names are taken from their predicted homology with *Arabidopsis thaliana* or rice. Sorghum gene IDs corresponding to each gene name in this figure are detailed in Additional file 6: Table S4

To find genes with missense mutations that may have a direct effect on expression, and hence possibly an effect on sugar accumulation, we isolated DEGs in each tissue that had at least one nonsynonymous change and were located in a region where PR22 had the BTx3197 allele (Fig. 5; Additional file 7: Tables S5, Additional file 8: Tables S6, Additional file 9: Tables S7). Among the top 50 differentially expressed genes with a high impact mutation (as predicted by snpEff) in the internode, one of the most interesting candidates is *SIP2* (Sobic.002G075800), which has a frameshift mutation in the Rio allele. *SIP2* has been shown to be an upstream regulator of carbohydrate metabolism in *Arabidopsis* [33], where individuals with a mutant *SIP2* allele produced less sugars. In Rio, *SIP2* is significantly downregulated during the vegetative stage compared to PR22, but significantly upregulated at all later stages, consistent with its putative role in increasing sugar metabolism and storage.

Another promising candidate is Sobic.009G235700 (labeled as “transporter” in Fig. 5), which has a predicted sugar transport domain with 4 amino acid substitutions differentiating Rio and BTx623 (Additional file 8: Tables S6). There are also 3 genes among the top 50 in internode with functions in cell wall metabolism: a pectin lyase, a pectinesterase (*PME61*), and a NAC transcription factor (*NAC032*). Recently, a mutation in another NAC gene was implicated as the causative variant underlying the *D* locus in sorghum, which differentiates dry and juicy-stalked varieties and has a large effect on sugar yield [34]. In the leaf tissue, the *SWEET3–6* transporter (labeled as *AtVEX1* in Fig. 5) was among the most highly differentially expressed genes, along with several bHLH transcription factors, which regulate many processes in plants [35]. Several members of the Myb and bZIP transcription factor families, which are also known for their roles in regulating plant development and response to abiotic factors [36], appear among the most differentially expressed genes in all tissue types, including the meristem (Fig. 5).

## Discussion

Overall comparison of the sweet and grain sorghum reference genomes revealed a high degree of collinearity and structural conservation. While this conservation appears to be in contrast to what has been observed among closely related varieties of maize [37], and is possibly even more conserved than what has been observed among rice lineages [13, 16], it should be noted that the two genotypes compared here do not represent the full spectrum of diversity among sorghums, and a comparison of a larger number of agronomically contrasting sorghum genotypes representing more historical differences will certainly reveal more structural differences. Among the few genes that have experienced expansions in Rio, most belong to a family of protein kinases with leucine rich receptor regions and could be under selection for differences in disease resistance between the two lines.

Among those genes that were deleted in Rio were several known members of the SWEET family, a group of sucrose transporters that have recently diversified in grasses, and include 23 distinct members in sorghum [20]. SWEETs are generally sucrose efflux transporters that move sugar from the source leaf into the phloem, but the specific functions of individual SWEET genes are more varied. A recent study exploring the SWEET activity in sorghum stems found a diverse range of temporal and spatial patterns across the entire gene family [21]. The fact that several of these transporters have been deleted in Rio could be indicative of a mechanism for retaining sugar in the stalk, rather than moving it into the seed as the final storage sink.

The importance of sugar transport in sorghum has been described in several other studies [7, 8, 19, 38]. Here, we also find several significantly differentially expressed sucrose transporters within each tissue type, along with many other differentially expressed transmembrane transporters and a large number of microtubule-related genes which may be responsible for their localization in the cell membrane. Further, our results indicated that many of the causal mutations may lie outside of the transport genes or their immediate upstream regions. Many of the significant changes in expression we observed occurred in genes with the same genetic background in both lines, and also coincided with time points when the level of soluble stalk sugars (Brix) was already at its highest.

It seems likely that many of these carbohydrate metabolism genes that show differential expression when both lines have the same allele are being regulated by the activity of other genes in the pathway and/or genetic differences at other locations in the genome. We observed an overall high level of nonsynonymous mutations in Rio, and two families of known post-translational regulators had several members with missense:silent polymorphism ratios > 1. These gene families have previously been shown to be associated with stress response, growth, and developmental pathways, including metabolite profile modulation [39], so it is possible that some members may be interacting with elements in the sugar metabolism pathway. Among those differentially expressed genes that had nonsynonymous mutations, we find a known sucrose transporter, *SWEET3–6,* along with *SIP2,* a gene shown to have an upstream role in sugar metabolism. Many of the other genes with predicted coding changes and significant differences in expression belonged to families of transcription factors that are known to have key roles in controlling plant secondary metabolism.

## Conclusions

Even though sweet sorghum is highly genetically similar to grain sorghum at the structural level, we find key differences in regulatory genes as well as potential deletions and loss-of-function mutations in sugar metabolism genes that are likely to play important roles in stem sugar accumulation. The reference genome we have generated for sweet sorghum will provide a useful resource for future agronomic and physiological studies by allowing researchers to better link underlying genetic architecture with observed changes in gene expression and plant phenotypes.

## Methods

### Rio reference genome

All Rio genetic material was obtained from a single seed source provided by W. Rooney at Texas A&M University. Sequencing was performed on a PacBio RS II system (Pacific Biosciences, Menlo Park, CA, USA) using 52 SMRT cell runs for a coverage of 75x of the genome. The genome assembly was constructed using FALCON [40] and polished with Quiver [41]. Homozygous SNPs and Indels were corrected using ~40X Illumina resequencing reads (2x250bp, 800 bp insert), and completeness of the final assembly was assessed by aligning genes from the existing *S. bicolor* reference at 90% identity and 85% coverage. Genome-guided transcript assemblies were made from close to 1 billion bp of 2x151bp paired-end Illumina RNAseq reads using PERTRAN (Shu, unpublished). PASA [42] alignment assemblies were constructed using the PERTRAN output from the Rio RNAseq data along with sequences from known *S. bicolor* expressed sequence tags (ESTs) associated with the current reference genome.

### Genome comparison and gene-gene alignment

MUMmer3.23 [43] was used to align the Rio reference genome to the latest version of the *S. bicolor* genome (v3.1.1) available from Phytozome [44]. To identify all rearrangements, including repeats, we ran *nucmer* with the following parameters: ‘--maxmatch, -c 200, -l 100 -b 200 -g 500’. The *nucmer* results were then uploaded to Assemblytics [45] to identify putative expansions and deletions with Rio. SNPs and small indels were annotated with snpEff [46]. snpEFF was also used to predict which SNPs had low, moderate, or high impacts. Homologous gene pairs were assigned as follows: the filtered coordinates file for all mapped MUMmer blocks was used to find the gene IDs contained within each block, and the 2 sets of genes were locally aligned using a Needleman-Wunsch algorithm [47] with a scoring matrix weighted by a BLAT [48] alignment similarity score calculated based on the transcript sequences for each gene. The same local alignment procedure was also used on all gene IDs located within regions called as either expansions or contractions by Assemblytics. Genes located within a MUMmer block but without a mapped homolog in the other genome were not considered as presence-absence variants (PAVs) or copy number variants (CNVs), since they seem most likely to be the result of annotation errors or a lack of transcription in one of the 2 genomes.

Rio genes not located within any MUMmer block were sorted into 2 groups: those with a BLAT score of at least 0.5 to at least one BTx623 gene, and those with no observed matches. Genes with a match were further sorted into those that matched a BTx623 gene that already had an assigned homolog in a MUMmer block, and those that matched an unpaired BTx623 gene. For genes with multiple matches, their score was weighted to take into account which pairings were also the most syntenic. Genes in one genome with no matches and no block assignments in the other were categorized as PAVs, while genes matching an already homologously paired gene were categorized as paralogous copies.

### Differential expression analysis

Material for RNAseq was collected at 6 biological stages, with 3 biological replicates for each sample (Additional file 4: Table S2). At every biological stage, tissue from the topmost fully developed leaf was harvested along with tissue from the topmost internode. During vegetative stages, meristems were isolated from within the topmost internode while floral and seed tissues were collected after plants had become reproductively active. All tissues were immediately placed into RNA Later at 4 °C, and then RNAs were subsequently extracted using the Qiagen RNeasy Plant Mini Kit plus DNase treatment. Libraries were run on a HiSeq 2500 with 2 × 150 reads. Individual quality filtered fastq files were trimmed using Trimmomatic v0.36 [49] to remove adapter sequences and low quality base pairs, then aligned to the Rio reference using TopHat v2.1.1 [50]. Read counts were calculated using HTSeq v0.6.1 [51] and DESeq2 [52] was used to find genes with a significant Genotype x Time interaction. Differentially expressed genes were clustered by expression pattern using EBSeqHMM. [53] GO enrichment analyses were performed on gene sets using the R package topGO [54]. Putative gene functions for differentially expressed genes were also inferred based on their homologous pairing with BTx623 genes described above.

### RIL breakpoint analysis

DNA was isolated from both PR22 and BTx3197 concurrently with the material grown for the Rio assembly. Sequencing was performed using a 2 × 250 paired end tight insert protocol on 1 lane of a HiSeq 2500 in Rapid Run mode. Raw Illumina reads from each of the 3 lines were filtered and trimmed using Trimmomatic v0.36 and then aligned to the Rio genome using Bowtie2 [55]. Mapped reads were filtered for PCR duplicates and sorted using Samtools v1.4 [56], and SNPs were called using the GATK v3.7 HalotypeCaller and GenotypeGVCFs tools [57]. The combined VCF file was filtered to remove sites with any missing genotype, a polymorphism between the aligned Rio reads and the Rio reference, or a heterozygous BTx3197 genotype. Further filtering was done to remove non-informative sites where both parents (Rio and BTx3197) had the same genotype. Informative SNPs were grouped into 15 SNP sliding windows (with no overlap), and the proportion of Rio:BTx3197 alleles was calculated for each window in PR22. Windows with a proportion greater than 2 were classified as Rio (R) haplotypes, while those with proportions < 0.25 were classified as BTx3197 (B) haplotypes, and breakpoints were identified wherever windows transitioned from R to B or vice versa.

## Additional files

Additional file 1: **Figure S1.** Frequency distributions of structural variants predicted by Assemblytics (based on Nucmer alignment of Rio and BTx623). In this analysis, BTx623 is the reference genome, so events such as insertions and deletions have occurred in Rio with respect to BTx623. (PDF 13 kb)

Additional file 2: **Figure S2.** Sugar accumulation in the topmost internode over time in the Rio and PR22 genotypes. Sugar is measured as Brix (soluble sugar concentration) in 3 biological replicates of each genotype at each of the 5 developmental time points used for the RNA seq analysis. The same plants were used for both Brix measurements and RNA material collection. (PDF 98 kb)

Additional file 3: **Table S1.** Known sucrose-related genes in sorghum and their associated gene IDs. (XLSX 10 kb)

Additional file 4: **Table S2.** Samples collected for RNA sequencing. (XLSX 10 kb)

Additional file 5: **Table S3.** SRA accession numbers and sample information. (XLSX 10 kb)

Additional file 6: **Table S4.** Gene IDs and descriptions for the top 50 DEGs with moderate to high impact mutations in each tissue. Only genes with the alternate allele in the RIL are considered. Genes correspond to those shown in Fig. 5. (XLSX 14 kb)

Additional file 7: **Table S5.** Predicted SNP effects for the top 50 Internode DEGs. Genes correspond to those shown in Fig. 5. Columns 4–31 are taken directly from the output of the program snpEff. Details can be found in the snpEff reference manual. Column 32 (GT.pvalue) is the significance of the Genotype x Time interaction term in the differential expression analysis. Columns 33–37 are the Fold Changes at each developmental stage, calculated as Rio/PR22 (Sweet/Non-Sweet). (XLSX 19 kb)

Additional file 8: **Table S6.** Predicted SNP effects for the top 50 Leaf DEGs. Genes correspond to those shown in Fig. 5. Details about columns are the same as described for Additional file 7: Table S5. (XLSX 19 kb)

Additional file 9: **Table S7.** Predicted SNP effects for the top 50 Meristem DEGs. Genes correspond to those shown in Fig. 5. Details about columns are the same as described for Additional file 7: Table S5. (XLSX 19 kb)

Additional file 10: **Table S8.** Predicted conserved domain mutations in protein sequences from the most differentially expressed genes. Genes are restricted to those with the alternate allele in the RIL. Only the top 50 DEGs in each tissue were considered. Only genes with at least one AA mutation in a conserved region are shown. Protein sequences from BTx and Rio were aligned with MUSCLE, using the msa R package from Bioconductor. CDD regions were predicted using the BTx sequences in NCBI’s batch web CD-Search tool (https://www.ncbi.nlm.nih.gov/Structure/bwrpsb/bwrpsb.cgi). Positions refer to the residue position within BTx. (XLSX 60 kb)

## Abbreviations

ANT: Anthesis
CNV: Copy Number Variant
DEG: Differentially Expressed Gene
EST: Expressed Sequence Tag
FL: Flag Leaf
GO: Gene Ontology
NB-LRR: Nucleotide Binding Leucine Rich Repeat
PAV: Presence Absence Variant
RI: Reproductive Initiation
RIL: Recombinant Inbred Line
SD: Soft Dough
SNP: Single Nucleotide Polymorphism
SPS: Sucrose Phosphate Synthase
SUS: Sucrose Synthase
SUT: Sucrose Transporter
V: Vegetative
