# Supplementary figures and images for: A new reference genome for Sorghum bicolor reveals high levels of sequence similarity between sweet and grain genotypes: implications for the genetics of sugar metabolism
Source: BMC Genomics. 2019 May 27;20:420. doi: 10.1186/s12864-019-5734-x (PMC6537160; doi:10.1186/s12864-019-5734-x)

# Variants 50 to 500 bp

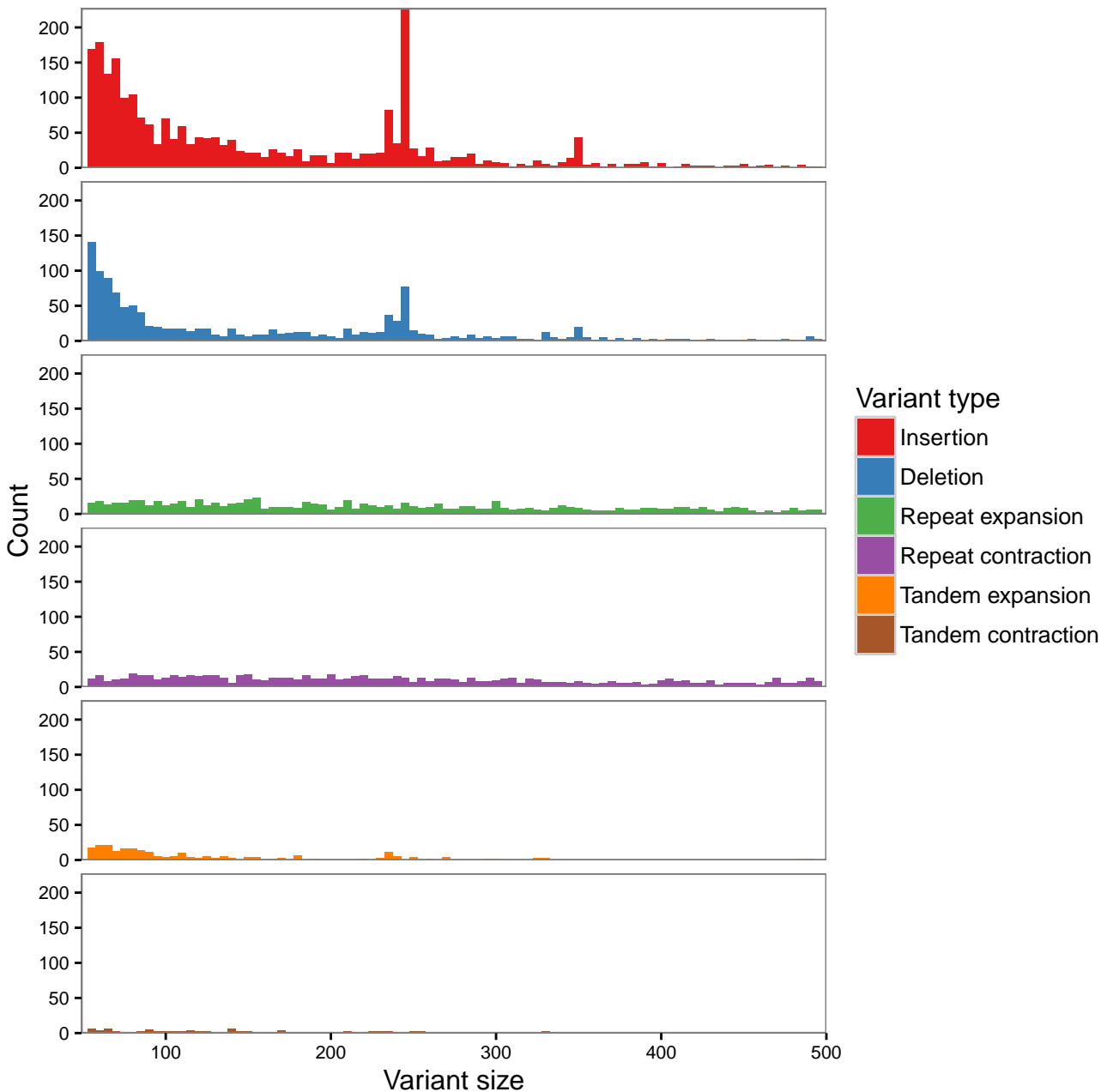

Supplement: Supplementary file 1 — Figure S1. Frequency distributions of structural variants predicted by Assemblytics (based on Nucmer alignment of Rio and BTx623). In this analysis, BTx623 is the reference genome, so events such as insertions and deletions have occurred in Rio with respect to BTx623. (PDF 13 kb) [file 12864_2019_5734_MOESM1_ESM.pdf]

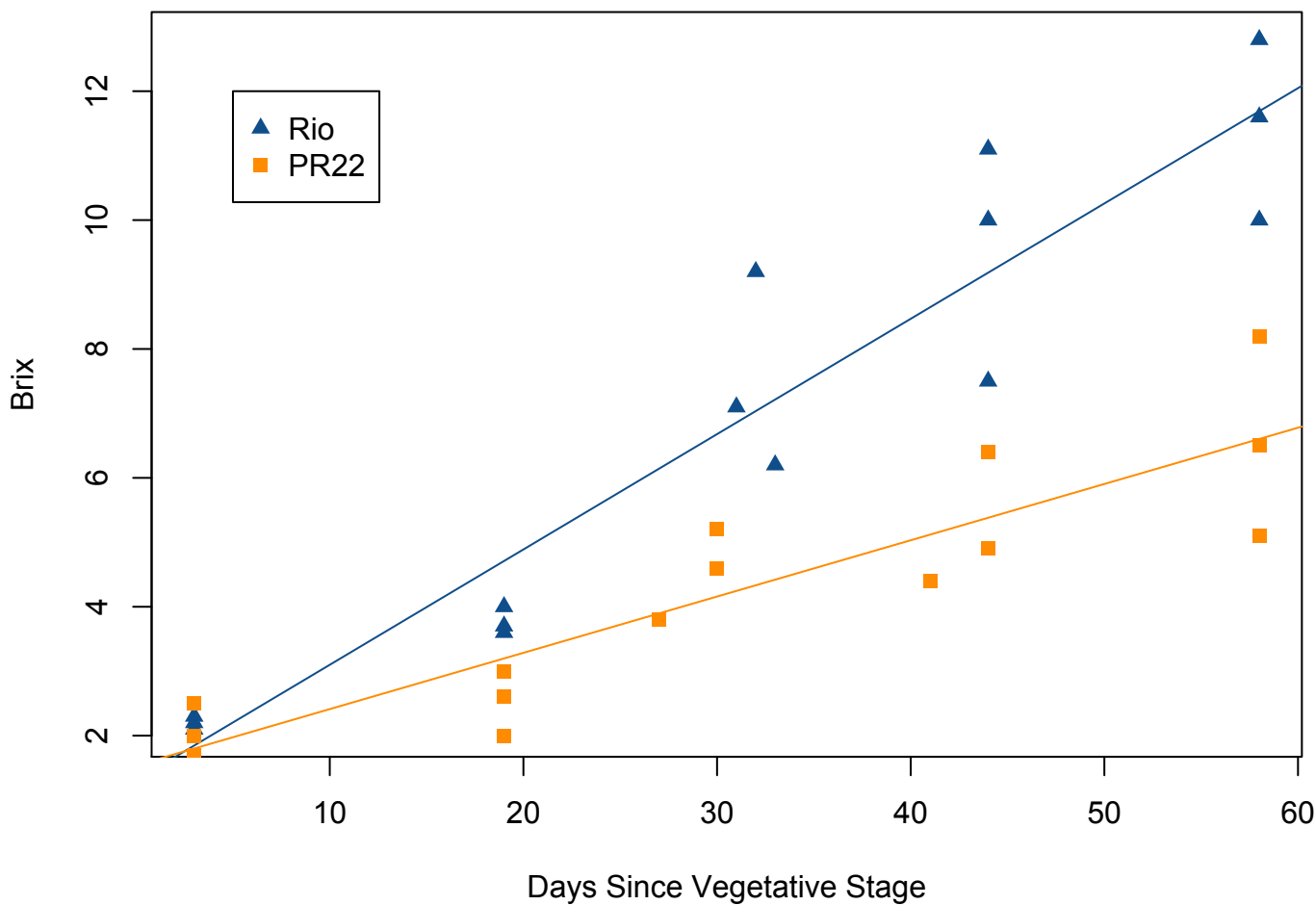

Supplement: Supplementary file 2 — Figure S2. Sugar accumulation in the topmost internode over time in the Rio and PR22 genotypes. Sugar is measured as Brix (soluble sugar concentration) in 3 biological replicates of each genotype at each of the 5 developmental time points used for the RNA seq analysis. The same plants were used for both Brix measurements and RNA material collection. (PDF 98 kb) [file 12864_2019_5734_MOESM2_ESM.pdf]
